# H_2_O_2_ mediates ALA-induced glutathione and ascorbate accumulation in the perception and resistance to oxidative stress in *Solanum lycopersicum* at low temperatures

**DOI:** 10.1186/s12870-018-1254-0

**Published:** 2018-02-15

**Authors:** Tao Liu, Xiaohui Hu, Jiao Zhang, Junheng Zhang, Qingjie Du, Jianming Li

**Affiliations:** 1 College of Horticulture, Northwest Agriculture & Forestry University, Yangling, Shaanxi 712100 China; 2 Key Laboratory of Protected Horticultural Engineering in Northwest, Ministry of Agriculture, Yangling, Shaanxi 712100 China

**Keywords:** 5-Aminolevulinic acid, Tomato, Chilling, Oxidative stress, Hydrogen peroxide, Redox state

## Abstract

**Background:**

Low temperature is a crucial factor influencing plant growth and development. The chlorophyll precursor, 5-aminolevulinic acid (ALA) is widely used to improve plant cold tolerance. However, the interaction between H_2_O_2_ and cellular redox signaling involved in ALA-induced resistance to low temperature stress in plants remains largely unknown. Here, the roles of ALA in perceiving and regulating low temperature-induced oxidative stress in tomato plants, together with the roles of H_2_O_2_ and cellular redox states, were characterized.

**Results:**

Low concentrations (10–25 mg·L^− 1^) of ALA enhanced low temperature-induced oxidative stress tolerance of tomato seedlings. The most effective concentration was 25 mg·L^− 1^, which markedly increased the ratio of reduced glutathione and ascorbate (GSH and AsA), and enhanced the activities of superoxide dismutase, catalase, ascorbate peroxidase, dehydroascorbate reductase, and glutathione reductase. Furthermore, gene expression of *respiratory burst oxidase homolog1* and H_2_O_2_ content were upregulated with ALA treatment under normal conditions. Treatment with exogenous H_2_O_2_, GSH, and AsA also induced plant tolerance to oxidative stress at low temperatures, while inhibition of GSH and AsA syntheses significantly decreased H_2_O_2_-induced oxidative stress tolerance. Meanwhile, scavenging or inhibition of H_2_O_2_ production weakened, but did not eliminate, GSH- or AsA- induced tomato plant tolerance to oxidative stress at low temperatures.

**Conclusions:**

Appropriate concentrations of ALA alleviated the low temperature-induced oxidative stress in tomato plants via an antioxidant system. The most effective concentration was 25 mg·L^− 1^. The results showed that H_2_O_2_ induced by exogenous ALA under normal conditions is crucial and may be the initial step for perception and signaling transmission, which then improves the ratio of GSH and AsA. GSH and AsA may then interact with H_2_O_2_ signaling, resulting in enhanced antioxidant capacity in tomato plants at low temperatures.

**Electronic supplementary material:**

The online version of this article (10.1186/s12870-018-1254-0) contains supplementary material, which is available to authorized users.

## Background

As an economically important crop, tomatoes (*Solanum lycopersicum* L.) are widely cultivated [1]. However, as a tropical and subtropical plant, tomato plant growth, development, and production are also negatively influenced by low temperatures (0 °C–15 °C) [2]. The low temperature (8 °C–15 °C) is widespread during the tomato production under protected cultivation in winter and early spring in China, which seriously restrict the normal production of tomatoes. Plants perceive and defend against the cold temperatures using a range of mechanisms, including the regulation of gene expression [3], redox state [4], and complex signaling [5, 6]. Beyond the limit of cold tolerance, reactive oxygen species (ROS) excessively accumulate [7]. ROS include hydroxyl radicals (·OH), superoxide anions (O_2_^−^), singlet oxygen (^1^O_2_) and hydrogen peroxide (H_2_O_2_) [8–10]. As strong oxidizers, high levels of ROS have pernicious effects that result in DNA damage, lipid peroxidation, protein denaturation, a decline in photosynthesis, enzyme activity impairment, and cell death [11, 12]. Consequently, maintaining moderate levels of ROS is essential in protecting against diverse abiotic and biotic stresses.

Maintaining a delicate balance between ROS generation and removal is important for plants, which is principally accomplished by antioxidant defence system [13, 14]. The main antioxidant defense system involves the ascorbate-glutathione (AsA-GSH) cycle, which consists of two dominating nonenzymatic antioxidants, reduced glutathione and ascorbate (GSH and AsA), and four enzymes [ascorbate peroxidase (APX), monodehydroascorbate reductase (MDHAR), dehydroascorbate reductase (DHAR) and glutathione reductase (GR)]. Together with these four enzymes, GSH and AsA reduce ROS via spontaneous biochemical reactions [8, 15]. In addition, superoxide dismutase (SOD) and catalase (CAT) also play key roles in antioxidant systems [16].

However, moderate oxidative stress and oxidative signaling are also essential to maintain plant growth and development [17–19]. For example, H_2_O_2_ generated by NADPH oxidases encoded by the *respiratory burst oxidase homologue1* (*RBOH1*) genes, play critical roles in tomato plant responses to oxidative stress as a signaling [20, 21]. ROS may also induce the regulation of groups of genes with protective functions [22], and crosstalk with endogenous phytohormones such as abscisic acid, gibberellins, salicylic acid, jasmonic acid, and ethylene, to regulate protective responses in plants under oxidative stress [4, 23]. The 5-aminolevulinic acid (ALA), an essential biosynthetic precursor of all tetrapyrrole compounds [24, 25], can also maintain moderate ROS levels via antioxidases and antioxidants [26–29]. However, few studies have focused on the interaction between H_2_O_2_ and cellular redox signaling in ALA-induced resistance to oxidative stress in plants at low temperatures.

In this study, low temperature perception and stress tolerance in both ALA-treated and untreated leaves of tomato plants were investigated by determining the roles and interactions of H_2_O_2_, glutathione, and ascorbate redox signaling induced by ALA at low temperatures.

## Methods

### Plant culture and experimental design

Tomato plants (cv. Jinpeng no. 1) sensitive to low temperature stress were used in this study. The seeds were germinated at 28 °C in petri dishes which lined with moistened filter paper. The germinated seeds were then sown in 50-well plates filled with a mixture of peat, perlite and vermiculite (2:1:1, *v*/v/v) and grown in a controlled environment greenhouse at Northwest Agriculture & Forestry University. The seedlings were transplanted into plastic pots (10 cm × 10 cm, one seedling per pot) containing the same mixture medium when the fourth true leaf was fully expanded. Plants were then placed in growth chambers at a temperature of 25 °C/18 °C (day/night), relative humidity of 65% ± 5%, and a photoperiod of 10.5 h [photosynthetic photonflux density (PPFD), 350 μmol·m^− 2^ s^− 1^] + 1.5 h (PPFD, 50 μmol·m^− 2^ s^− 1^)/12 h (day/night). Each plant was irrigated once every two days with 100 mL distilled water and fertilized at every other occasion with 100 mL of a 50% concentration of Hoagland nutrient solution. The experiments began when the fifth true leaves were completely expanded, and a total of 45 plants for each treatment were analyzed.

To explore the effects of ALA on plant tolerance to low temperature, tomato plants were first sprayed with distilled water (control) or 0, 1, 5, 10, 25, 50, or 100 mg·L^− 1^ ALA (Sigma Aldrich, St. Louis, MO, USA) solution. The selection of ALA concentrations was based on the studies of Korkmaz et al. [30], Ali et al. [31] and our preliminary experiments. Each plant was treated with 6 mL (according to our preliminary experiment results) of ALA solution or distilled water on both sides 1.5 h before the night, and a few drops of surfactant (Tween 20) were added to enhance adherence. Twelve hours later, the control plants were still under normal conditions as previously mentioned, while other plants were subjected to low temperature at 15 °C/8 °C (day/night) under the same light and humidity regime as previously mentioned. After 24 h, the degree of stress tolerance was assessed by measuring changes in the net photosynthetic rate (Pn), malondialdehyde (MDA) content, and relative electrical conductivity (REC), and the plant growth indexes were measured after 6 days. With the same processing time and conditions as mentioned above, the H_2_O_2_ content, maximal quantum yield of PSII photochemistry (Fv/fm), and the antioxidase and antioxidants were determined in plants treated with distilled water or 25 mg·L^− 1^ ALA under normal condition (Control and ALA) or low temperature (LT and LT + ALA).

A level of 25 mg·L^− 1^ ALA was used in the following experiments. To study the effects of H_2_O_2_ on plants induced by ALA, the plants were treated with distilled water or 25 mg·L^− 1^ ALA under normal conditions. The transcripts of *RBOH1* and levels of H_2_O_2_ were then measured during the next 24 h.

To determine the roles of H_2_O_2_, GSH, and AsA in ALA-induced tolerance against oxidative stress at low temperatures, the tomato leaves were pretreated with 5 mM dimethylthiourea (DMTU, a H_2_O_2_ and O_2_^−^ scavenger) [32, 33], 100 μM diphenyleneiodonium (DPI, an inhibitor of oxidative burst and NADPH oxidases which generates H_2_O_2_) [2, 32], 1 mM buthionine sulfoximine (BSO, an inhibitor of GSH biosynthesis) [32, 34], or 1 mM acriflavine (AF, an inhibitor of AsA biosynthesis). After 8 h, the leaves were sprayed with 25 mg·L^− 1^ ALA, 5 mM H_2_O_2_ [35], 5 mM GSH, or 1 mM AsA. Twelve hours later, the plants were exposed to low temperatures. After 24 h low temperatures, the MDA content and REC were measured.

In addition, we applied the compound inhibitors to further examine the interaction between H_2_O_2_, GSH, and AsA with the same treatments and index measurement as described above.

### Measurement of plant growth indexes

After the height and stem diameter of each plant were measured, the plants were washed with distilled water, topical moisture was removed, and the plants were dissected into shoots and roots. The fresh weights of dissected tissues were determined, and then the dry weights were obtained after drying for 15 min at 105 °C and then 75 °C for 72 h. There were seven independent biological replicates for each independent treatment, and three independent experiments were performed.

### Measurement of Pn, Fv/fm, MDA, and REC

Pn was measured with a portable photosynthesis system LI-6400 (LI-COR Inc., USA). Fv/fm was measured according to the methods of Pérez-Bueno et al. [36] with the Open FluorCam FC 800-O and analyzed using the Fluorcam7 software (PSI, Brno, Czech Republic). The level of membrane lipid peroxidation in leaves was valuated by measuring the MDA content as described by Hodges et al. [37]. The REC was measured according to Zhou and Leul [38].

### Measurement of H_2_O_2_ content

The H_2_O_2_ content in tomato leaves was estimated according to the method of Willekens et al. [39]. Mixed 800 mL extracted sample with 400 mL reaction buffer (pH 4.4) containing 4 mM 2, 2′-azino-di (3-ethylbenzthiazoline-6-sulfonic acid) and 100 mM potassium acetate, 400 mL deionized water and 0.25 U of horseradish peroxidase (HRP). The H_2_O_2_ content was determined by measuring the absorbance at 412 nm.

Histochemical staining of O_2_^−^ was carried out as described by Jabs et al. [40] and the histochemical staining of H_2_O_2_ was fulfilled according to the methods of Thordal- Christensen et al. [41].

### Measurements of antioxidant enzyme extracts and activities

For the measurement of SOD (EC 1.15.1.1) activities, one unit of SOD activity was defined as the amount of enzyme needed 50% inhibition of the decrease of nitro blue tetrazolium (NBT), as monitored at 560 nm [42].

CAT (EC 1.11.1.6) activity was measured by monitoring the decreases of H_2_O_2_ at 240 nm for 2 min. DHAR (EC 1.8.5.1) activity was assayed by monitoring the changes of ascorbate at 265 nm for 3 min. GR (EC 1.6.4.2) activity was assayed by monitoring the decrease of NADPH at 340 nm for 3 min. APX (EC 1.11.1.11) activity was assayed by monitoring the decreases of ascorbate at 290 nm for 2 min. MDHAR (EC 1.6.5.4) activity was assayed by monitoring the decreases of NADPH at 340 nm for 3 min. The methods above were described according to Noctor et al. [35].

### Measurements of glutathione and ascorbate levels

The glutathione content was assayed by monitoring the changes of 2-nitro-5-thiobenzoic acid absorbance at 412 nm for 5 min. The ascorbate content was assayed by monitoring the changes of ascorbate at 265 nm for 5 min [35].

### RNA extraction and qRT-PCR analyses

Total RNA was extracted from tomato leaves using the Plant RNA Kit (OmegaBio-Tek, Doraville, GA, USA) according to the supplier’s instructions. The total RNA was then reverse-transcribed using a PrimeScript TM RT reagent kit with a gDNA Eraser (Takara, Shiga, Japan), following the manufacturer’s instructions. The gene specific primers of *RBOH1* for qRT-PCR were as follows; (forward, 5′-CGGAACAGGCAACGGTGTA-3′; reverse, 5′-TGCGAAATCGGAACGATAAA- 3′) and for the actin gene were (forward, 5′-GGGATGGAGAAGTTTGGTGGTGG-3′; reverse, 5′-CTTCGACCAAGGGATGGTGTAGC-3′), which was used as an internal control.

### Statistical analysis

All data were analysed with SAS 8.0 software (SAS Institute, Cary, NC, USA) using Duncan’s multiple- range test at a significance level of *P* < 0.05.

## Results

### The effects of ALA concentrations on tomato membrane lipid peroxidation, Pn, and growth under low temperature stress

**Fig. 1:**
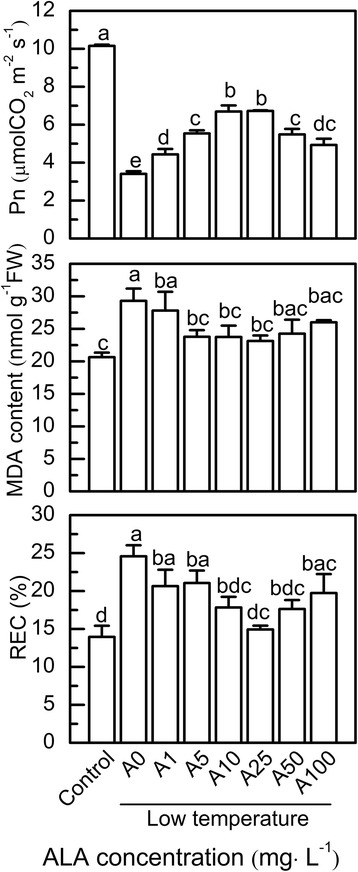
ALA alleviation of low temperature-induced oxidative stress was dose dependent. Data are expressed as the mean ± standard error of three independent biological replicates. Different letters above the bars indicate a significant difference of *P* < 0.05

Compared to the control, low temperatures increased the levels of both MDA and REC, and decreased the level of Pn, while ALA treatment at low concentrations (10–25 mg·L^− 1^) dramatically decreased the levels of both MDA and REC, and increased the level of Pn at low temperatures. At the optimal concentration of 25 mg·L^− 1^ ALA, MDA and REC were 21.1% and 39.3% lower, respectively, and Pn was 97.5% higher than that of plants not treated with ALA under low temperature (A0) (Fig. 1). In addition, treatment with ALA significantly improved the plant growth of tomatoes compared with A0 plants (Additional file 1: Table S1). The protective effect of ALA against low temperature stress was attenuated when treated with ALA concentrations either higher or lower than 25 mg·L^− 1^ (Fig. 1).

### The effects of ALA on the *RBOH1* expression and H_2_O_2_ accumulation, and Fv/fm in tomato plants under low temperature stress

**Fig. 2:**
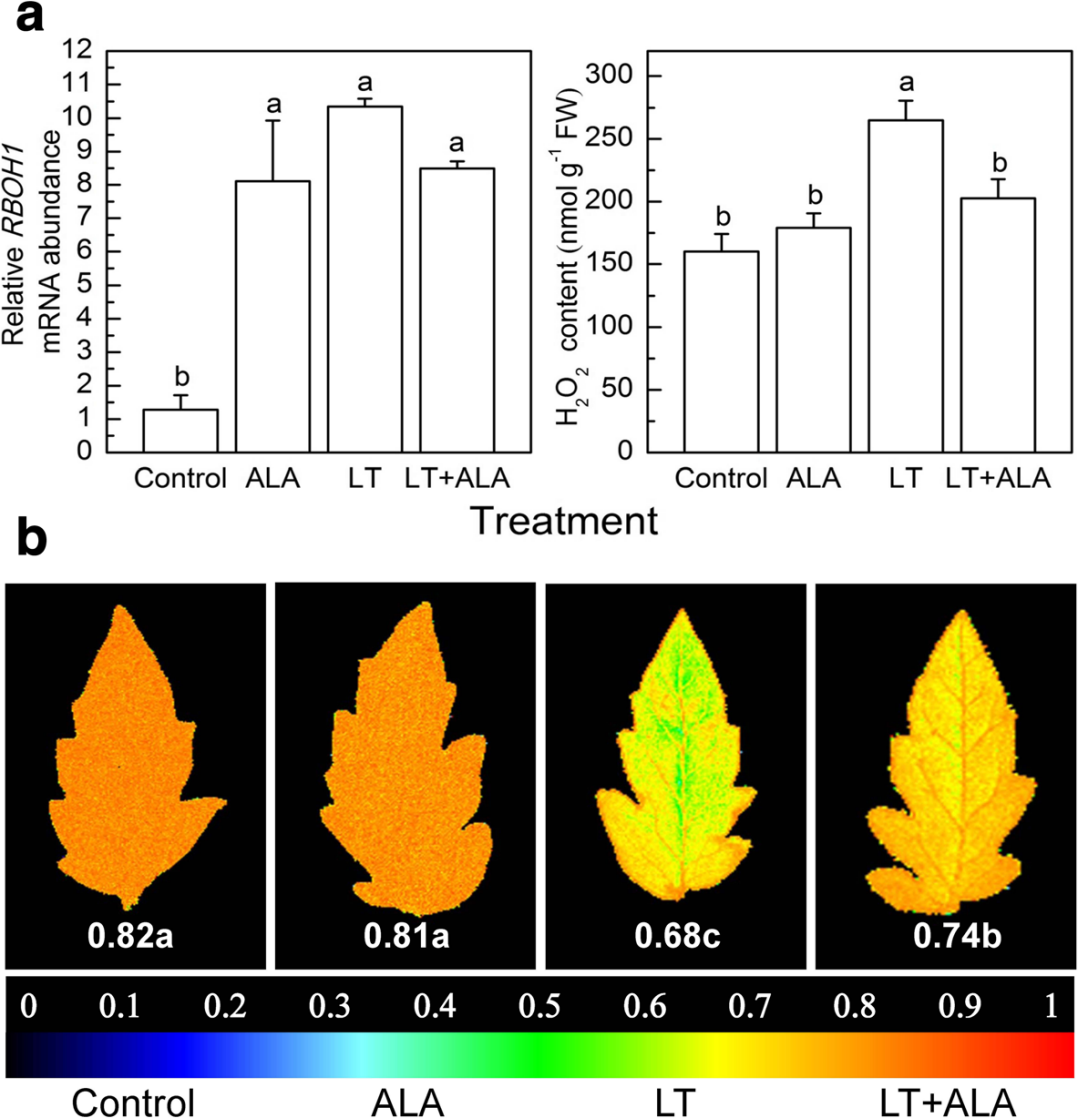
ALA reduced the *RBOH1* transcription and H_2_O_2_ levels, and improved the Fv/fm at low temperature. **a** *RBOH1* transcription levels (the levels in control plants at 0 h was normalized as 1) and H_2_O_2_ content; **b** Images of the Fv/fm, the false color code depicted at the bottom of the image ranges from 0 (black) to 1(red). Data are expressed as the mean ± standard error of three independent biological replicates. Different letters above the bars indicate a significant difference at *P* < 0.05

Under control conditions, ALA treatment upregulated the *RBOH1* expression and increased the H_2_O_2_ content of plants (Fig. 2a). Low temperature increased the *RBOH1* expression and the level of H_2_O_2_, and resulted in the highest levels among all treatments (Fig. 2a), while the Fv/fm significantly declined (Fig. 2b). However, treatment with ALA at low temperatures reduced *RBOH1* expression and H_2_O_2_ content by 17.9% and 23.5%, respectively (Fig. 2a), while the level of Fv/fm increased by 8.8% (Fig. 2b), as compared to the untreated low temperature-stressed plants (LT).

Compared with the control, ALA treatment had no distinct effect on O_2_^−^ and H_2_O_2_ histochemical staining, but low temperatures caused a remarkable accumulation of O_2_^−^ and H_2_O_2_ in leaves and mesophyll cells, which were alleviated by ALA treatment at low temperatures (LT + ALA) (Additional file 2: Figure S1).

**Fig. 3:**
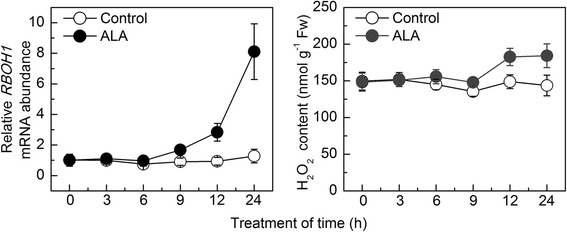
ALA induced upregulation of *RBOH1* transcription levels and accumulation of H_2_O_2_ content under normal conditions. The *RBOH1* transcription levels in control plants at 0 h was normalized as 1. Data are expressed as the mean ± standard error of three independent biological replicates

To verify the roles of H_2_O_2_ in plants induced by ALA, plants treated with ALA under normal conditions for 12 h showed an upregulation of *RBOH1* transcripts and H_2_O_2_ content of 203.0% and 22.7%, respectively (Fig. 3).

### The effects of ALA on antioxidation in tomato plants under low temperature stress

**Fig. 4:**
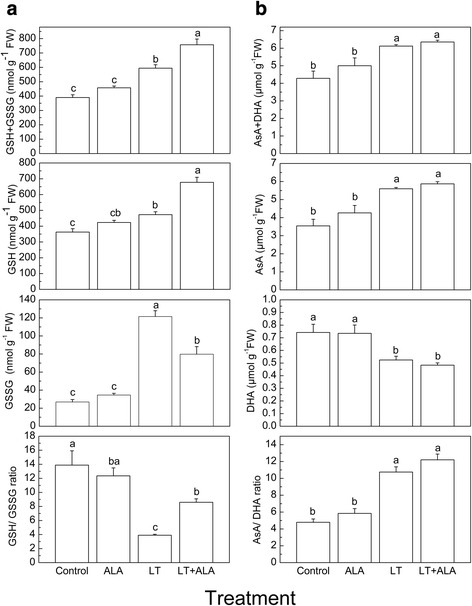
ALA regulated the redox status of glutathione and ascorbate to inhibit low temperature-induced oxidative stress. **a** Content of GSH + GSSG, GSH, and GSSG, and the ratio of GSH/GSSG; **b** Content of AsA + DHA, AsA, and DHA, and the ratio of AsA/DHA. Data are expressed as the mean ± standard error of three independent biological replicates. Different letters above the bars indicate a significant difference at *P* < 0.05

Under normal conditions, ALA treatment had no significant effects on the antioxidases and antioxidants (Fig. 4, Table 1). However, low temperature stress significantly enhanced the activities of SOD, APX, MDHAR, and DHAR, and the ratio of AsA/DHA, while the activities of CAT and GR and the ratio of GSH/ GSSG were greatly reduced as compared to the control (Fig. 4, Table 1). Treatment with ALA at low temperatures significantly increased the activities of SOD, APX, DHAR, and GR, and the ratio of GSH/GSSG by 14.8%, 42.6%, 33.9%, 113.5%, and 120.3%, respectively, but decreased the activity of MDHAR by 23.2% compared with untreated low temperature-stressed plants (LT) (Fig. 4, Table 1).

**Table 1:** The activities of antioxidant enzymes in tomato leaves induced by ALA

Treatment	SOD activity (unit mg^− 1^ prot)	CAT activity (μmol mg^− 1^ prot min^− 1^)	APX activity (μmol mg^− 1^ prot min^− 1^)	GR activity (nmol mg^− 1^ prot min^− 1^)	MDHAR activity (nmol mg^− 1^ prot min^− 1^)	DHAR activity (nmol mg^− 1^ prot min^− 1^)
Control	8.12 ± 0.33c	105.72 ± 7.72a	1.70 ± 0.12c	62.97 ± 7.38ba	393.72 ± 42.09b	198.14 ± 6.73c
ALA	8.73 ± 0.56cb	98.56 ± 3.96ba	1.62 ± 0.17c	70.35 ± 9.33a	397.26 ± 45.58b	213.81 ± 17.61c
LT	9.92 ± 0.32b	76.43 ± 6.69c	2.35 ± 0.28b	20.2 ± 1.84c	615.68 ± 29.78a	461.11 ± 27.8b
LT + ALA	11.39 ± 0.48a	85.57 ± 2.73bc	3.34 ± 0.14a	43.13 ± 4.34b	472.68 ± 43.14b	617.55 ± 48.94a

Data are expressed as the mean ± standard error of three independent biological replicates. Different letters indicate a significant difference at *P* < 0.05

### The effects of H_2_O_2_, GSH, and AsA on ALA-induced antioxidation on tomato plant tolerance to low temperature stress

**Fig. 5:**
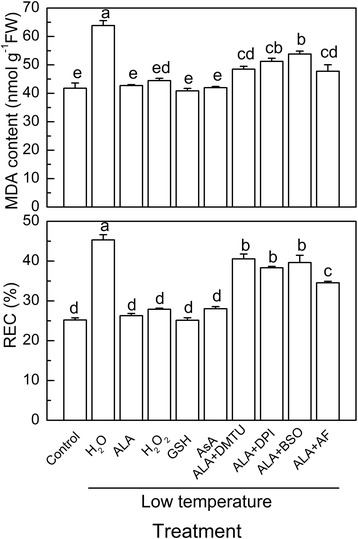
Involvement of H_2_O_2_, GSH, and AsA in ALA-induced oxidative stress tolerance at low temperatures. Data are expressed as the mean ± standard error of three independent biological replicates. Different letters above the bars indicate a significant difference at *P* < 0.05

At low temperatures, application of H_2_O_2_, GSH, or AsA dramatically reduced the MDA content by 30.3%, 36.0% and 34.2%, respectively, and decreased REC by 38.5%, 44.5%, and 38.1%, respectively, compared with the application of H_2_O in tomato plants (Fig. 5). However, pretreatment of the tomato leaves with DMTU, DPI, BSO, or AF mostly eliminated the ALA-induced tolerance to oxidative stress under low temperature (Fig. 5).

**Fig. 6:**
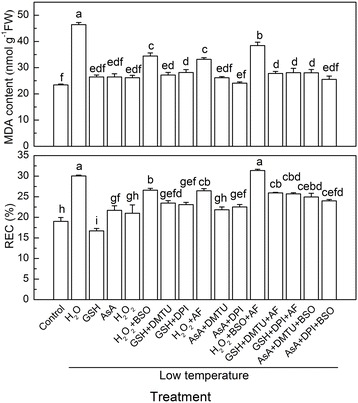
The relationships among H_2_O_2_, GSH, and AsA in the inhibition of low temperature-induced oxidative stress. Data are expressed as the mean ± standard error of three independent biological replicates. Different letters above the bars indicate a significant difference at *P* < 0.05

To further characterize the interactions among H_2_O_2_, AsA, and GSH in defending against low temperature stress, the effects of BSO, AF, DMTU, and DPI on H_2_O_2_, AsA, and GSH-induced low temperature tolerance were determined. Pretreatment with BSO or AF largely reduced the alleviated effect of H_2_O_2_ on the oxidative stress-induced increase of MDA levels and REC at low temperatures (Fig. 6). In contrast, pretreatment with DMTU or DPI partially blocked the GSH- or AsA-induced oxidative stress tolerances at low temperatures (Fig. 6).

## Discussion

### Exogenous ALA induces defense against low temperature-induced oxidative stress in tomato plants

Many studies showed that application of high concentrations of ALA could induce the generation of free tetrapyrroles, such as protochlorophyllide (Pchlide). Meanwhile, free tetrapyrroles generated highly reactive singlet oxygen which was the trigger of severe oxidative stress [25, 43]. In contrast, low concentrations of ALA alleviated oxidative stres**s** in many crops, such as peppers [30], soybeans [44], melons [45] and oilseed rape [46]. In the present study, the results showed that low temperatures caused severe oxidative stress by generating O_2_^−^ that was converted to H_2_O_2_, while pretreatment with ALA alleviated the oxidative damages from the low temperature stress (Additional file 2: Figure S1). Notably, the effects of ALA on coping with oxidative stress and improving plant growth were much weaker at concentrations above or below 25 mg·L^− 1^ (Fig. 1, Additional file 1: Table S1). This might be due to the dual effects of ALA that caused ROS accumulation with excessive free tetrapyrroles [43], or removed ROS by antioxidation [29], suggesting that the mode of action of ALA on plants was dose dependent.

### The AsA-GSH cycle plays key roles in ALA-induced defenses against oxidative stress at low temperatures

Plants accumulating free tetrapyrroles can induce photooxidative damage [25]. However, there is no evidence to suggest that ALA directly scavenges ROS (O_2_^−^ and H_2_O_2_). The reduction of ROS by ALA might therefore be dependent on reactions of antioxidant systems. O_2_^−^ could be rapidly converted into H_2_O_2_ by SOD [12], which could then be converted to H_2_O or O_2_ by a GSH and/or a AsA regenerating cycle and/or CAT [47]. In the present study, GSH and AsA levels and the activities of some key enzymes (APX, GR, and DHAR) involved in the AsA-GSH cycle and SOD were dramatically increased, the activity of CAT was only slightly enhanced, and the activity of MDHAR was significantly decreased with ALA treatment under low temperature compared with untreated low temperature-stressed plants (LT) (Table 1, Fig. 4). These results suggested that O_2_^−^ was reduced to H_2_O_2_ by SOD, which was then scavenged by the CAT and the AsA-GSH cycle induced by ALA. With AsA as a substrate, APX played a direct role, and the AsA regeneration was mainly catalyzed by increased activity of DHAR, but not MDHAR, from DHA to AsA [47]. Overall, the results showed that the AsA-GSH cycle induced by ALA eliminated excessive H_2_O_2_ in tomato plants at low temperatures.

### The interaction between H_2_O_2_ signaling and the glutathione and ascorbate redox states is essential for ALA-induced oxidative stress perception and tolerance at low temperatures

It is well-known that ROS plays critical roles in mediating signal transmission in plants [48]. The upregulation of *RBOH1* expression induced by ALA caused H_2_O_2_ generation under normal conditions (Fig. 3) [20], suggesting that ALA might induce plant perception and self-protective mechanisms via intensive H_2_O_2_ signaling, which subsequently alleviated the oxidative stress at low temperatures.

Regarding peroxide metabolism and their regeneration, GSH and AsA are interdependently associated [49]. Our studies showed that treatment with GSH and AsA induced tomatoes tolerance to oxidative stress at low temperatures, while pretreatment with AF and BSO largely blocked the ALA-induced resistance against oxidative stress (Fig. 5). Cellular GSH and AsA are reductants involved in the defense against ROS, and are also known to affect signaling intensities, as well as transmitting information from environmental stresses to their respective targets (stress-related genes, phytohormone levels, or regulatory proteins) [49–51]. Pretreatment with ALA might therefore induce initial defense mechanisms via H_2_O_2_ signaling and the interaction with other signaling compounds, such as GSH and AsA [4, 52], to induce resistance to oxidative stress at low temperatures [7]. In addition, GSH is a transmitter of intracellular ROS signaling, while enough of the AsA pool and a high AsA/DHA ratio might play a minor role in ROS signaling via the GSH redox cycle [53], and via phytohormones, and gene expressions [49]. The results therefore suggested that the increases in GSH and AsA were essential for H_2_O_2_-induced tolerance to low temperature-induced oxidative stress (Fig. 6).

Taken together, intensive H_2_O_2_ signaling induced by pretreatment with ALA may mediate the redox status of glutathione and ascorbate to perceive the oxidative stress and subsequent resistance at low temperatures. In addition, GSH and AsA may interact with H_2_O_2_ signaling and other phytohormones, to induce a series of relevant gene expressions, resulting in an enhanced antioxidant capacity in tomato plants at low temperatures.

## Conclusions

Exogenous ALA alleviated the low temperature-induced oxidative stress in tomato plants via an antioxidant system, and the most effective concentration of ALA was 25 mg·L^− 1^. Pretreatment with ALA under normal conditions may induce initial plant perception and signaling transmission via elevated H_2_O_2_ signaling, which then activates defense mechanisms by glutathione and ascorbate redox signaling, inducing plant resistance to oxidative stress at low temperatures. In addition, glutathione and ascorbate redox signaling may interact with H_2_O_2_ to regulate the protective responses.

## Additional files

Additional file 1: **Table S1.** The effects of ALA concentrations on the growth of tomato seedlings under low temperature stress. Data are expressed as the mean ± standard error of seven independent biological replicates. Different letters indicate a significant difference at *P <* 0.05. (DOCX 15 kb)

Additional file 2: **Figure S1.** Histochemical staining of the effects of ALA on ROS accumulation. O_2_^−^ (**a**) and H_2_O_2_ (**b**) in tomato leaves and mesophyll cells. The photographs were obtained using an Olympus motorized system microscope (BX51, Olympus, Tokyo, Japan) at 1000× magnifications. Bar = 20 μm. (JPEG 3465 kb)

## Abbreviations

AF: Acriflavine
APX: Ascorbate peroxidase
AsA: Reduced ascorbate
BSO: Buthionine sulfoximine
CAT: Catalase
DHA: Dehydroascorbate
DHAR: Dehydroascorbate reductase
DMTU: Dimethylthiourea
DPI: Diphenyleneiodonium
Fv/fm: Maximal quantum yield of PSII photochemistry
GR: Glutathione reductase
GSH: Reduced glutathione
GSSG: Oxidized glutathione
H_2_O_2_: Hydrogen peroxide
MDHAR: Monodehydroascorbate reductase
O_2_^−^: Superoxide anions
Pn: Net photosynthesis rate
ROS: Reactive oxygen species
SOD: Superoxide dismutase
