# Supplementary material for: H2O2 mediates ALA-induced glutathione and ascorbate accumulation in the perception and resistance to oxidative stress in Solanum lycopersicum at low temperatures
Source: BMC Plant Biol. 2018 Feb 15;18:34. doi: 10.1186/s12870-018-1254-0 (PMC5815209; doi:10.1186/s12870-018-1254-0)
Supplement: Supplementary file 1 — Table S1. The effects of ALA concentrations on the growth of tomato seedlings under low temperature stress. Data are expressed as the mean ± standard error of seven independent biological replicates. Different letters indicate a significant difference at P < 0.05. (DOCX 15 kb) [file 12870_2018_1254_MOESM1_ESM.docx]

Table S1.

| Treatment | Plant height  (cm) | Stem diameter (cm) | Shoot | | Root | |
| --- | --- | --- | --- | --- | --- | --- |
|  |  |  | Fresh weight (g) | Dry weight (g) | Fresh weight (g) | Dry weight (g) |
| Control | 26.27±0.79a | 5.22±0.08a | 12.01±0.99a | 0.72±0.02a | 1.98±0.08a | 0.19±0.01a |
| A0 | 17.57±0.52d | 4.54±0.09d | 6.08±0.11b | 0.62±0.02bdc | 1.53±0.08c | 0.15±0.01b |
| A1 | 18.47±0.47dc | 4.73±0.06cbd | 6.20±0.06b | 0.62±0.03bdc | 1.60±0.05cb | 0.16±0.01b |
| A5 | 19.23±0.64dc | 4.76±0.12cbd | 6.42±0.07b | 0.67±0.02bdac | 1.61±0.07cb | 0.16±0.00b |
| A10 | 19.57±0.57c | 4.87±0.06cb | 7.13±0.08b | 0.68±0.03bac | 1.69±0.05cb | 0.17±0.00b |
| A25 | 22.23±0.64b | 4.93±0.04b | 7.28±0.11b | 0.69±0.03ba | 1.76±0.05b | 0.17±0.00ba |
| A50 | 19.07±0.43dc | 4.75±0.12cbd | 6.81±0.16b | 0.64±0.02bdc | 1.70±0.05cb | 0.17±0.00b |
| A100 | 18.00±0.52dc | 4.65±0.06cd | 6.07±0.11b | 0.60±0.03dc | 1.56±0.09cb | 0.15±0.01b |
