# Supplementary figures and images for: H2O2 mediates ALA-induced glutathione and ascorbate accumulation in the perception and resistance to oxidative stress in Solanum lycopersicum at low temperatures
Source: BMC Plant Biol. 2018 Feb 15;18:34. doi: 10.1186/s12870-018-1254-0 (PMC5815209; doi:10.1186/s12870-018-1254-0)

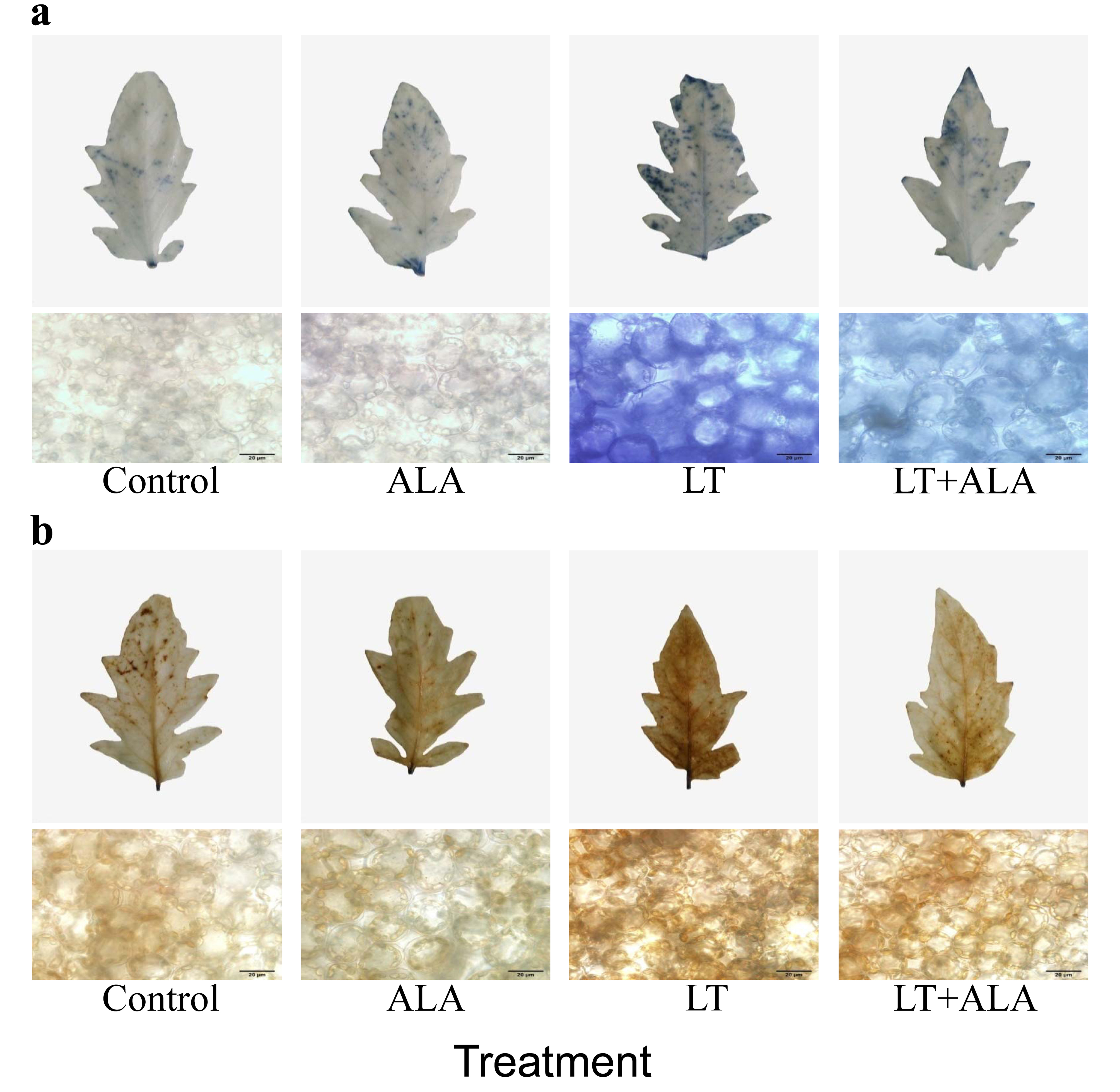

Supplement: Supplementary file 2 — Figure S1. Histochemical staining of the effects of ALA on ROS accumulation. O2− (a) and H2O2 (b) in tomato leaves and mesophyll cells. The photographs were obtained using an Olympus motorized system microscope (BX51, Olympus, Tokyo, Japan) at 1000× magnifications. Bar = 20 μm. (JPEG 3465 kb) [file 12870_2018_1254_MOESM2_ESM.jpg]
